# Hepatitis E Virus Seroprevalence among Men Who Have Sex with Men, United Kingdom

**DOI:** 10.3201/eid1902.121174

**Published:** 2013-02

**Authors:** Brendan A.I. Payne, Manjul Medhi, Samreen Ijaz, Manoj Valappil, Emma J. Savage, O. Noel Gill, Richard Tedder, Ulrich Schwab

**Affiliations:** Author affiliations: Newcastle-upon-Tyne Hospitals, Newcastle-upon-Tyne, UK (B.A.I. Payne, M. Medhi, M. Valappil, U. Schwab);; Newcastle University, Newcastle-upon-Tyne (B.A.I. Payne);; Health Protection Agency, London, UK (S. Ijaz, E.J. Savage, O.N. Gill, R. Tedder)

**Keywords:** hepatitis E, hepatitis E virus, viruses, homosexuality, male, seroepidemiologic studies

**To the Editor:** Immunosuppression might be associated with chronic carriage of hepatitis E virus (HEV) (*1*,*2*). HIV-infected persons could be at increased risk for HEV acquisition (*3*). If HIV infection is a risk factor for HEV, the risk will probably be mediated by associated behavioral factors. Men who have sex with men (MSM) are known to be at risk for transmission of enteric infection (*4*). Because of increasing prevalence of chronic liver disease induced by various causes among HIV-infected persons, it is necessary to determine whether these patients are at risk for HEV acquisition and possible hepatic decompensation (*5*).

We aimed to establish the contribution of HIV infection and MSM to seroprevalence of HEV among banked serum specimens. We used an unlinked, anonymous HIV seroprevalence survey of sexual health clinic attendees in England, Wales, and Northern Ireland, compared results from testing of residual serum samples collected for routine syphilis testing from sentinel clinics, and analyzed basic epidemiologic data (*6*). We examined serum samples collected during a 3-year period (2006–2008) and stored at −80°C. All samples were from male patients, 20–44 years of age. IgG against HEV was measured by using ELISA (Wantai; Fortress Diagnostics, Antrim, UK). To further increase the specificity for a seroprevalence analysis, and in accordance with previous work (*7*), we defined only samples with an optical density/cutoff value ≥1.5 as reactive and those in the range 1.0–1.5 as weakly reactive.

We analyzed 422 serum samples collected during 2008, comprising 146 samples from MSM with positive HIV test results, 135 from MSM with negative HIV test results, and 141 from heterosexual men with negative HIV test results. Thirty (7.1%) serum samples showed IgG reactivity against HEV and 3 (0.7%) additional samples showed weak reactivity. We examined the effect of HIV infection on prevalence of IgG against HEV by comparing samples from HIV-infected MSM with those from HIV-negative MSM . Seroprevalence rates did not differ significantly (HIV-positive MSM 7.5%; HIV-negative MSM 10.4%; p = 0.4). 

We then examined the effect of being MSM as a risk factor for HEV infection. Prevalence of IgG against HEV among HIV-negative heterosexual men was 3.5%, significantly lower than that among MSM (odds ratio 3.1, p = 0.025, for comparison with non-HIV–infected MSM). We examined the relationship of status of IgG against HEV among MSM to the presence of an acute non-HIV sexually transmitted infection (STI) at the time of serum sampling. No association was found (acute STI, 14 [9.1%] of 154 vs. no acute STI, 11 [8.7%] of 127; p = 0.9). Similarly, no statistical association was found between HEV antibody status and the location of the clinic that provided the serum sample (London, 21 [10.0%] of 211; United Kingdom excluding London, 4 [5.7%)] of 70; p = 0.3). As has been observed for the general UK population (*7*), we observed a trend toward increasing prevalence of antibodies against HEV with patient age (20–34 y, 9 [6.3%] of 142; 35–44 y, 16 [11.5%] of 139), although this trend did not reach significance (p = 0.13). Our samples were from persons who were younger than the previously described cohort of UK persons with increased prevalence of antibodies against HEV (born before approximately1960) (*7*). Multivariate analysis with the above variables showed that MSM (p = 0.044) and age group (p = 0.026) were independently associated with HEV seroprevalence. To explore recent temporal trends in HEV seroprevalence among MSM, we examined serum samples from 977 MSM collected during the 3-year study period. We observed an unexpected association between antibody prevalence and year of serum collection (2006, 4 [2.1%] of195; 2007, 26 [5.2%] of 501; 2008, 26 [9.3%] of 281; p = 0.003 (Figure).

**Figure:**
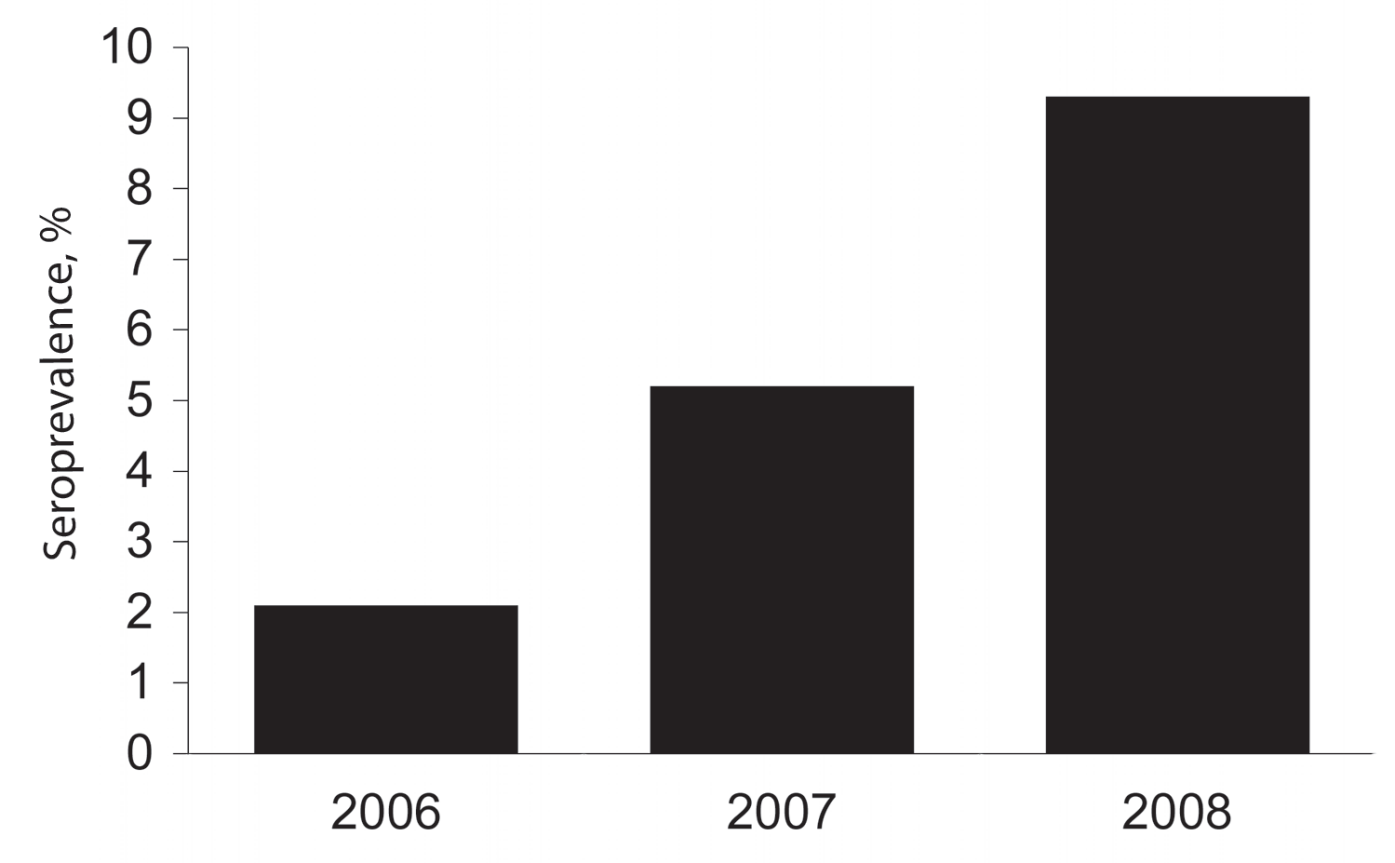
Hepatitis E virus seroprevalence among men who have sex with men, United Kingdom, 2006–2008.

We provide evidence that MSM might be at risk for HEV acquisition and confirm that HIV infection does not appear to be a risk factor. Although our study is of moderate size, and we have limited epidemiologic data owing to its unlinked, anonymized nature, the fact that patient groups are drawn from the same clinics should minimize the effect of unrecognized confounding factors. The pathologic mechanisms for HEV acquisition among MSM may plausibly include oro–anal sexual practices, which have been implicated in recent outbreaks of *Shigella flexneri* infection in this group (*8*). That ano–genital transmission of HEV is unlikely is supported by our finding that prevalence of antibodies against HEV was not more common among patients with an acute STI.

The shift in prevalence of antibodies against HEV among MSM occurred while HEV activity in the United Kingdom was increasing (*9*,*10*). The routes of transmission of indigenously acquired HEV infection in industrialized countries remain a subject of investigation, but our observations suggest that activity among MSM could expose this group to increased transmission. Thus, the putative combination of increased exposure in the general UK population and increased transmission among MSM suggests that HEV incidence and seroprevalence could increase for this group in the near future and couldbecome a substantial public health problem.
